# Social Marketing of Electronic Coupons Under the Perspective of Social Sharing Behavior

**DOI:** 10.3389/fpsyg.2021.746928

**Published:** 2021-10-20

**Authors:** An Shin Shia, Kuei-Feng Chang, Yu-Huang Huang

**Affiliations:** ^1^School of Business, Lingnan Normal University, Zhanjiang, China; ^2^School of Management, Guangzhou University, Guangzhou, China; ^3^Department of Marketing Management, Central Taiwan University of Science and Technology, Taichung, Taiwan

**Keywords:** electronic coupons, shared marketing, social sharing behavior, consumer behavior, marketing psychology

## Abstract

This study aimed to (1) explore the correlated influential factors that promote consumers' sharing behaviors of electronic coupons, (2) identify relevant knowledge gaps on e-coupon sharing, and (3) develop a theoretical model to explain the e-coupon sharing behavior of consumers. This study suggested that familiarity and professionalism of the referral source have significant and positive impacts on the feeling of credibility of e-coupons, which promote consumers' willingness to share. Moreover, this research used electronic coupons as the product from (1) the concept of social marketing and (2) the perspective of the social sharing behavior of consumers. The research findings of this study showed that (1) community-based social marketing is covered in the social norm as social individuals frequently hope to share benefits with others. (2) People's sharing purpose is to promote their established social relationship via sharing the benefits to others. In this case, benefits referred to advantageous products' attributes and values consumers found on the products such as credibility of the products. (3) The interlaced display of products and social psychology in the field of social marketing was also discussed in this study. In short, the research results of this study theoretically contributed to indicate the connection mechanism between (A) social benefit-sharing behavior and (B) product benefits. That is to say, the feeling of credibility and willingness to shares are covered by the social benefit-sharing behavior mechanism, while the product-benefit mechanism covers professionalism, feeling of credibility and willingness to share. Note that the (A) social benefit-sharing behavior mechanism works in the form of social norms in sharing benefits to an individual's social networks; the (B) product-benefit mechanism work in the form of product values and attributes.

## Introduction

With the popularity of social media, it has become a trend for businesses to use it to promote their brands and products; and electronic coupons (e-coupons) are one of the most important marketing methods. E-coupons can be used to attract coupon-prone consumers, increase product visibility, and encourage repeat (Li et al., 2019). Due to the cross-spatial nature of communication (Huang and Tang, 2016), broadness, and precise targeting features such as portability, e-coupons are considered to have great commercial value (Banerjee and Yancey, 2010). Users participate in the marketing process through social media by sharing preferential information (Li et al., 2019) about products while playing the important binary role of marketer and ordinary user (Jansen et al., 2009). When consumers get an electronic coupon through various social media, they can use it themselves or forward it to others by reposting it on social media. The trust established between the recipient and the reposter increases the likelihood of the e-coupon and related information to be accepted by the recipient. Therefore, there is also an increased chance of the recipient becoming a potential consumer. In this way, consumer relationships on social networks have become vital in disseminating e-coupons.

In short, the product discussed in this study is electronic coupons via the fundamental concept of social marketing; the authors expect to use the perspective of social sharing behavior of consumers to comprehensively explain that: the community-based social marketing is actually behind the spirit of social norms. That is to say, social individuals expect to share benefits with the others in order to maintain their existing social network via sharing of products' benefits and values. This scenario discussed in this study is innovative. The components for study are products, but the behavioral phenomenon under discussion is commonly social good's sharing behavior - social marketing of advantageous products' attributes and values. On the other hand, it can also be viewed that consumers have discovered products' values and attributes, which is identified as the credibility of the products in this study, and based on learned social norms in terms of maintaining existing social networks, consumers share the benefits to the other potential consumers. Therefore, in the conclusion of this article, we once again propose an innovative concept to distinguish the display and classification of products and social psychology in the field of social marketing. It is expected that such an advanced explanation of social marketing behavior can assist the extension of social marketing theory.

## Literature Review

### E-Coupon

The coupon is an effective marketing tool for merchants to conduct price promotions; coupons are often considered a win-win application (Ferrer-Gomila et al., 2018). It can effectively attract price-prone consumers, increase product visibility, and encourage them to make repeat purchases (Ren et al., 2021). E-coupon (e-coupon) is also one important marketing instrument specifically for B2C e-commerce. In order to increase the exchange rate of e-coupons and reduce marketing costs, it is important to relocate customers who have received e-coupons and have a higher redemption tendency (Li et al., 2020). Through various electronic media and disseminated and used on major social media sites, e-coupons' format includes cash vouchers, experience vouchers, gift vouchers, discount vouchers, special coupons, redemption vouchers, and shopping red envelopes. In short, coupons, a mainstay of marketing for decades, but their short- and long-run effects on sales are still not understood fully (Reimers and Xie, 2018), which leads authors of this study for further in-depth exploration on its influential impacts, focusing on the sharing behaviors of e-coupons.

### Social Marketing Theory

As social marketing is a new emerging topic in recent years, scholars are still looking for defining its influential antecedents, process and output (efficiency and effectiveness measurement) variables, and ways of systematic approach (Saunders and Truong, 2019; Truong et al., 2019) for extending this theory. Unlike traditional marketing that focuses on profitability, social marketing often uses social media to achieve the so-called “commonly beneficial to social good goal” in the form of structural social-behavior change programs *via* combinative methods of social science and marketing approach (Truss, 2010) to provide solutions to potential social issues (French and Gordon, 2015). Therefore, social marketing's components are unavoidable to discuss products, such as e-coupon mentioned in this study, and consumer orientation (Lefebvre et al., 1988) such as influential antecedents of this study (professionalism, familiarity, and credibility) of individuals' behavioral pattern, basing on the social benefit-sharing intention of consumers.

Social marketing has a certain degree of influence on society, such as social health promotion, as well as the attention on the sustainability of social marketing (Saunders et al., 2015), mental health problem prevention, environmental psychology (McKenzie-Mohr, 2000), youth problems (Lefebvre, 2013), and the like. Using community-based social marketing (CBSM) skills that include both social norms and commitment, environmental psychologist McKenzie-Mohr (2000), except information distribution, perceptual barriers also need to be conquered to trigger behavioral change and thus to speed up social change efficiency.

#### Shared Marketing

The concept of shared marketing is based on this social network relationship. Shared marketing combines emotional factors with product information sharing. New consumers use product recommendations or evaluations from other consumers that they trust to understand the product better. In this way, it is easier for the new consumer to trust the product and conduct product purchase activities. The trust in this product recommendation comes from its source, who is either a reliable individual familiar with the product or an expert with a good reputation in the field, rather than the merchant who issues the coupon (Cheung and Lee, 2012). According to Drugǎu-Constantin (2019), the top four consumers' purchase decision influencers include (1) recommendations from a friend, family. and known acquaintance (37%); (2) TV ads (17%); (3) online review or recommendation from someone within your social media circle (16%), (4) online review by someone you do not know in real life (10%). From Drugău-Constantin's research in 2019, we can see that the most popular referral influencers are familiar friends, family, and online reviews from someone you know or do not know. Merchants take advantage of fast-growing social media and use shared marketing to provide e-coupons for certain incentives, such as price discounts or special offers, to target consumer groups (Li et al., 2019) and encourage them to share among their acquaintance circles. With e-coupons, companies can get good publicity at lower costs.

As an important part of the classic 4P marketing theory, a promotion strategy is a means for companies to advertise the advantages of their products and persuade consumers to make purchasing behavior (Kotler and Amstrong, 1998). As a widely used promotion method, online marketing often uses word-of-mouth marketing, viral marketing, etc., to spread awareness or increase one consumer's willingness to buy the company's products (Hill et al., 2006). The basic idea behind it is to create a cascading effect to spread product marketing to social networks further, that is, from one person to another through social circles (Dawood and Shahrokh, 2019). The characteristics of viral information transmission are fundamentally related to its content and its psychological impact on consumers' willingness to share information with others (Heath et al., 2001). Belk (2007) argued that sharing is a means of acquiring and consuming goods, in which “two or more people can enjoy the benefits of owning an item.” According to Bowman and Narayandas (2001) people tend to choose someone close to them to share a particular content. For example, Hollowell et al. (2019) analyzed the most commonly used platforms by the share of generational groups and concluded that the gig economy is beneficial for everyone. When consumers choose to share information about products and services with others, they strengthen their relationship with the recipient (Frenzen and Nakamoto, 1993; Aral and Van Alstyne, 2011). This information transmission method, which combines emotional factors and product information sharing, establishes the basic form of shared marketing. The relationship between online communities allows marketing information to be communicated, making it easier to obtain a response from the recipient of information.

### Social Sharing Behavior of Benefit

Studies on coupons and coupon effectiveness have emerged substantially. The sharing economy promotes innovative ways of getting involved in a community (Sutherland and Jarrahi, 2018; Graessley et al., 2019) and facilitates sharing routines (Deka, 2018). Therefore, researchers must come up with a better model that conveys effective ways of making couponing a profitable marketing strategy. One approach is to identify consumers' behavioral models in enhancing the effectiveness of coupon promotions, such as through sharing and discussion.

According to some literature on social psychology (Petty et al., 1995; Eagly and Kulesa, 1997), consumers often discuss topics relevant to their benefits (self-participation and self-involvement) which develops attitude elaboration. Social media helps shape corporate brand reputation (Bratu, 2019); sharing e-coupons through social media sites such as Facebook, Line, and WeChat fit into this type of behavioral model. By sharing e-coupons, individuals can satisfy the needs of their friends (Berger et al., 2020) and families in obtaining benefits and building up relationships and strong emotional connections. Most previous literature has focused on direct mail, newspapers, and direct delivery of coupons (Krishna et al., 1989; Inman and McAlister, 1994); and very few have investigated the effects of the sharing behavioral model of consumers.

This study aims to identify the factors that influence and promote consumers' sharing behaviors of e-coupons, recognize the relevant knowledge gaps in e-coupon sharing that need to be addressed, and develop a theoretical model incrementally increase the literature about e-coupon sharing behavior of consumers.

#### Sharing Benefit as the Social Norm

Consumers' sharing intention of coupon is related to social behavior such as social norms (Shimp and Kavas, 1984), e.g., spousal approval for coupon usage and feelings of being a “smart shopper,” and is targeted at increasing the consumer's internal reference price (Schindler, 1974). That is to say; consumers increasingly share e-coupons with their relatives, family, and friends to satisfy each other's need to obtain benefits from social networks as part of social norms. Social marketing theory application remains scarce in social marketing. Therefore, with three studies (in three different contexts: walking to and from school, binge drinking, and fruit and vegetables packed into lunchboxes), David et al. (2020) demonstrated TPB measures' applications in social marketing theory in detail - TPB measure conceptualized that intention is determined by attitudes, subjective norms (SNs) and perceived behavioral control (PBC) toward the behavior, which is the core concept of the famous TPB theory (Theory of Planned Behavior; Fishbein and Ajzen, 2011).

#### Motivation for Sharing E-Coupons

Many factors motivate individuals to share information; the most common include doing the right thing, conveying goodwill, informing others, or offering benefits or rewards to others (Lewis et al., 2005). Motivation for sharing can be described as the user's behavior of sharing obtained information to others to meet specific needs or desires, follow and associate the information needs of others, and establish a channel of communication with others for sharing of information. More than 80% of reposters' sharing behavior can be attributed to altruistic sharing motivations, and the beneficiaries of shared e-coupons, aside from others, can also be themselves. The e-sharing platform tends to favor all users (Querbes, 2018), in which social and emotional values are instrumental in driving individuals to reassess businesses (Zhang et al., 2018; Meilhan, 2019). Thus, individuals can meet the needs of friends (Berger et al., 2020) and family members in terms of gaining benefits, building relationships, and strong emotional connections.

#### E-Coupon Recipient's Familiarity With the Referral Source and Their Willingness to Share E-Coupons

Compared to paper coupons, e-coupons are distributed by merchants and through third-party platform integration distribution or friend recommendation. The distribution of e-coupons through the use of social networks on social media has become more widespread and more accurate and has gradually attracted the attention of merchants. At the same time, the academic community is also paying attention to researches related to understanding the relationship between the person who recommended the e-coupon and the recipient. Miricǎ (2019) adopted Neuroscientific methods to inspect and found that consumer's deliberate sense-making ability to acquire a higher degree of persuasion bottomed on instinctive feedback in terms of interest mechanisms. That is to say, because of the combination of familiarity and professionalism, consumers sharing e-coupon with others is already a kind of interesting behavior, which may lead to the benefits of relationship enhancement of sharers. The degree of closeness of the relationship between two parties in information communication can be divided into strong connection and weak connection. Chan et al. (2009) found that consumers' social relationships in their communities determine how they receive and share information and their willingness to act. Also, De Bruyn and Lilien (2008) pointed out that when consumers receive shared recommendations from close friends or family members who are strongly connected, the possibility of accepting the information is greater than when it comes from unfamiliar individuals. This communication method based on social relations has a stronger transmission ability and effect than traditional methods. Therefore, this study proposes the following:

H1: The familiarity of the e-coupon's recipient with the referral source positively impacts the recipients' willingness to share e-coupons.

#### Relationship Between the Professionalism of the E-Coupon's Referral Source and the Recipients' Willingness to Share E-Coupons

Professionalism refers to the source's knowledge of the e-coupon and information related to it and the ability to provide information with a high degree of matching with user information needs and expectations. Goldberg and Hartwick (1990) defined professionalism as having professional knowledge of the information being shared and providing accurate information. Previous studies have mostly focused on the effects of professionalism. For instance, Bansal and Voyer (2000) noted that when consumers collect opinions, they are more willing to get advice from experts than non-experts and are more susceptible to the influence of expert opinions. Also, Sweeney et al. (2013) mentioned that when opinion leaders appear to be more professional as sources of information, their effect on audience behavior is more significant. A source that provides professional opinions and suggestions is more likely to affect consumers' willingness to accept the information and perform the behavior. Therefore, this study proposes the following:

H2: The professionalism of the e-coupon's referral source has a positive impact on the recipient's willingness to share e-coupons.

### The Relationships Among Familiarity, Professionalism, the Feeling of Credibility, and Recipients' Willingness to Share E-Coupons

#### The Relationship Between Familiarity With the Referral Source and the Feeling of Credibility of the E-Coupon

As social media becomes more integrated into people's daily lives, disseminating information has become simpler; however, the propensity of information has become a problem. People have to select and filter information to obtain only the necessary ones, and the most important criterion for filtering information is the feeling of credibility of the source (Belkin, 1978); Trust is vitally important in creating and maintaining successful long-term relationships between organizations and consumers (Pop et al., 2021). Hovland and Weiss (1951) defined the feeling of credibility as the trustworthiness of the information source, which is largely determined by the receiver's dependability and professional interpretation of the information source. Tseng and Fogg (1999) referred to the feeling of credibility as the receiver's subjective cognition of the information and the subjective evaluation of trust in the source of information during the process of information dissemination.

Differences in online information source familiarity can have different effects on consumers' feelings of credibility. According to Bowman and Narayandas (2001), compared with the promotional text posted on merchants' websites, the dissemination of information among consumers generated more expectations for knowledge exchange, attracted more attention, and developed consumer trust. Therefore, trust in the company or merchant developed through relationships among consumers is stronger than the one between merchants and consumers. The trust effect of recommendations from strangers and friends is different; the stronger the relationship between the source and the recipient of information, the higher the feeling of credibility of the information (Wirtz and Chew, 2002). When information comes from peers rather than marketers, consumers give more trust and value to the information sent to them. Therefore, this study proposes the following:

H3: The familiarity with the referral source has a positive impact on the feeling of credibility of the e-coupon.

#### The Relationship Between the Professionalism of the Referral Source and the Feeling of Credibility of the E-Coupon

The feeling of credibility of the information also depends on the subjective judgment of the recipient as to whether the referral source has expertise. The characteristics and the level of expertise of the reposter on the e-coupon being shared can greatly affect the quality of information transmission. Popescu and Ciurlǎu (2019) indicated that trust, which can be built by online reviews, is the key feature for sharing, and personal reputation helps validate the prearrangement of the commodity. Personal reputation is referred to as a qualification of professionalism to recommend commodities in this study. Also, Bansal and Voyer (2000) stated that the higher the level of expertise of the sender, the greater the dependence and trust of the receiver on the message; thus, the higher the level of professionalism of the e-coupon's sender, the higher the degree of matching of the provided information with the receiver and the more likely the receiver will believe the accuracy of the information, thereby increasing trust on the e-coupon and the information it contains. Therefore, this study proposes the following:

H4: The professionalism of the referral source has a positive impact on the feeling of credibility of the e-coupon.

#### The Relationship Between the Feeling of Credibility and the Recipients' Willingness to Share E-Coupons

Related research showed that the feeling of credibility could have a significant impact on one's willingness to share. In other words, the trustworthiness of the information and the information sources are important criteria for determining whether people are willing to try or make efforts to accept the information. The results of Sledgianowski and Kulviwat (2009) showed that the willingness of social network users to share is significantly affected by the trustworthiness of the information. Teng et al. (2014) noted that the level of feeling of credibility of the source can ultimately be linked to the confidence and acceptance of the recipient of the message. The results of the study by McKnight et al. (2002) on user's trust in online sellers showed that user trust led to the adoption of seller's suggestions.

On the contrary, users' distrust of referral sources caused the refusal of the referred information content. For e-coupons, the higher the feeling of credibility of the referral source and the information being shared, the less likely it is for people to refuse it, and the more likely it is to be accepted, used, or shared. Therefore, this study proposes that:

H5: The feeling of credibility of e-coupons has a positive effect on the recipients' willingness to share e-coupons.

**Figure 1:**
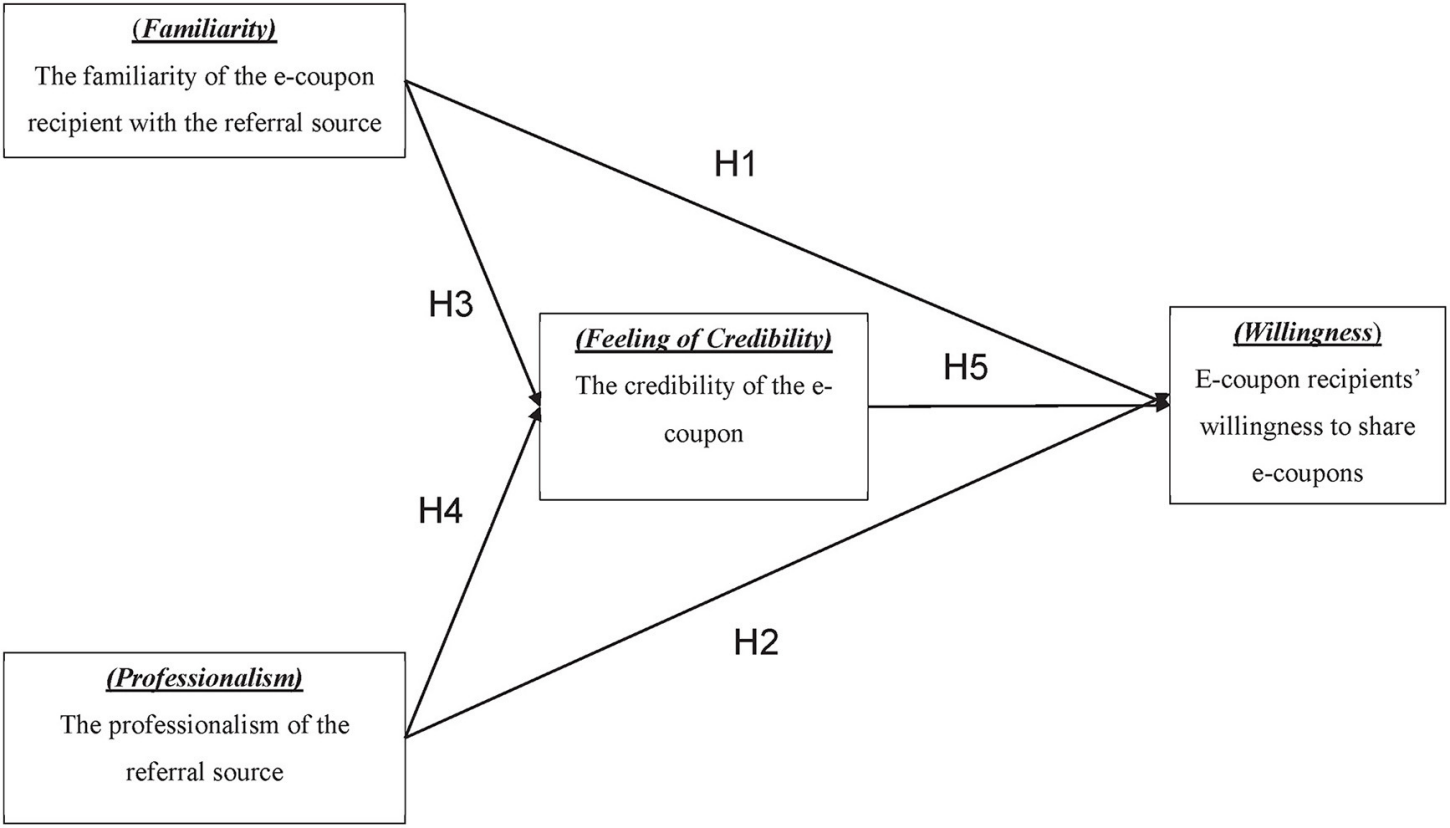
Research framework of this study.

Figure 1 illustrates the research framework of this study. The correlations between variables are based on the related literature review.

## Methodology

This research collected data through survey questionnaires. The structural equation model (SEM) analysis was performed to determine the relationship between variables, and the SPSS and AMOS software were utilized to verify the hypotheses.

### Descriptive Statistics of Sample Structure

The respondents' data collected in this study include the respondents' gender, age, educational level, personal monthly income, monthly frequencies in using or sharing e-coupons, etc.

This study was conducted in a convenient sampling method. A total of 210 questionnaires were distributed in Guangzhou, China, of which 200 were valid questionnaires, and the effective response rate was 95.2%. There are many universities located in Guangzhou Province in China. Although respondents are mostly college students, the range and scope of users' sharing e-coupons are connected with relationships, which cover all parts of the country. The detailed information of the sample structure is shown in Table 1.

**Table 1:** Description on the characteristics of research objects.

**Items**		**E-coupon users**	
		**Number of** **people**	**%**
Gender	Male	80	40%
	Female	120	60%
Age	Age 18 years and below	26	13%
	19–24 years old	167	83.50%
	25–34 years old	5	2.50%
	35–44 years old	–	–
	45 years old and above	2	1%
Educational level	Technical secondary school/senior high school and below	1	0.50%
	College/Undergraduate	196	98%
	Postgraduate and above	3	1.50%
The monthly disposable amount (RMB)	1,000 RMB and below	27	13.50%
	1,001–2,000	149	74.50%
	2,001–3,000	19	9.50%
	3,001 and above	5	2.50%
Daily average online time	2 h and below	6	3%
	2–4 h	40	20%
	4–6 h	70	35%
	6–8 h	57	28.50%
	More than 8 h	27	13.50%
Average number of e-coupons used per month	0 times	21	10.50%
	1–2 times	78	39%
	3–4 times	50	25%
	5–6 times	21	10.50%
	7 times and above	30	15%

### Questionnaire Design and Measurement

The 200 participants were asked to rate their experiences and behaviors in utilizing and sharing e-coupons using a 7-point Likert scale (1 represents strongly disagree and 7 represents strongly agree). The survey questionnaire was pre-tested first, and the analysis results showed that it had good reliability and validity. The pre-tested questionnaire was then developed into a formal questionnaire which was administered during the research proper. A total of 200 valid questionnaires out of the 210 that were distributed (effective recovery rate was 95.2%).

Each variable contains 5 items (Table 2). Items were formed from the referred source according to the different Asian cultures in terms of questioning; the referred sources are from information and concepts adopted from Fishbein and Ajzen (1975), Salwen (1987), Frenzen and Davis (1990), and Gilly et al. (1998). Operational definitions of each variable include: Familiarity (The user's understanding and communication with the recommender of the e-coupon); Professionalism (My e-coupon sharer is a professional in the field involved in e-coupons and has good cognitive insights about the e-coupon); Credibility (I think the recommended e-coupon can be trusted); Willingness to Share (The referee's willingness to accept, use or recommend and share e-coupons).

**Table 2:** Questionnaire content.

**Variable**	**Operational definition**	**Items**
Familiarity	The user's understanding and communication with the recommender of the e-coupon	1. I know the person who shares the e-coupons with me.
		2. I have frequent contacts with the person who shares the e-coupons with me.
		3. I will talk about the little things in life to the person who shares the e-coupons with me.
		4. I will share life experiences with the person who shares the e-coupons with me.
		5. I will express my true feelings to the person who shares the e-coupons with me.
Professionalism	My e-coupon sharer is a professional in the field involved in e-coupons and has good cognitive insights about the e-coupon.	1. I believe that sharer of the e-coupon understands the field involved in the e-coupon
		2. I believe that sharer of the e-coupon has the professional knowledge of the field involved in the e-coupon
		3. I believe that sharer of the e-coupon has experience in the field covered by the e-coupon
		4. I believe that sharer of the e-coupon has relevant capabilities in the field covered by the e-coupon
		5. I believe that sharer of the e-coupon has good knowledge of the field involved in the e-coupon
Credibility	I think the recommended e-coupon can be trusted.	1. I think the recommended e-coupon contains a complete information
		2. I think the recommended e-coupon information is reliable
		3. I think the recommended e-coupons are not biased
		4. I think the recommended e-coupons are trustworthy
		5. I think the recommended e-coupon information is true
Willingness to Share	The referee's willingness to accept, use or recommend and share e-coupons	1. I will accept the information of this e-coupon
		2. I think this e-coupon is worth using
		3. If the budget allows, I will use the e-coupon
		4. I am willing to share the e-coupon to other friends
		5. I would like to recommend a friend to use the e-coupon

*Items were formed from the referred source according to the different Asian cultures in terms of questioning; the referred sources are from information and concepts adopted from Fishbein and Ajzen (1975), Salwen (1987), Frenzen and Davis (1990), and Gilly et al. (1998)*.

### Analysis

#### Analysis of the Measurement Model

The AMOS 23.0 was used to conduct a confirmatory factor analysis to test the validity of the scale utilized in this study. According to the recommendations of Bagozzi and Yi (1988), the three most commonly used indicators for verifications are individual item reliability, potential variable combination reliability (CR), and average variation extraction (AVE). If the individual item reliability or the factor load is >0.5, the CR is >0.6, and the AVE is >0.5, then the scale has good aggregation validity. Also, if AVE is greater than the square of the correlation coefficient of the same variable, then the questionnaire has high convergence and discriminant validity. The analysis results of this study met the above criteria, indicating that the questionnaire has good reliability, convergence validity, and discriminant validity. Related information is shown in Table 3.

**Table 3:** Differential validity analysis and variable correlation coefficient.

	**Familiarity**	**Professionalism**	**Feeling of** **credibility**	**Willingness** **to share**
Familiarity	0.892			
Professionalism	0.124	0.848		
Feeling of credibility	0.345***	0.340***	0.783	
Willingness to share	0.243***	0.286***	0.544***	0.743
C.R.	0.951	0.927	0.887	0.856
AVE	0.795	0.719	0.612	0.552
Cronbach's α	0.951	0.927	0.889	0.861

*Inter-correlation coefficient is below the diagonal, and the square root of AVE estimates are presented diagonally*.

* P ≤ 0.05; ** P ≤ 0.01;

*** *P ≤ 0.001*.

#### Hypotheses Verification

The SEM was used to analyze and explore the relationship between the variables to verify the research hypotheses (H1–H5) in this study. The model fit indicators are shown in Table 4. The results showed that the data fit the model well and that the goodness of fit of each index reached acceptable standards. Therefore, the model of this study is acceptable.

**Table 4:** Model fit indicators and reference value.

**Model fit indicators**	**CMIN/** **DF**	**GFI**	**NFI**	**IFI**	**TLI**	**CFI**
Acceptable standards	<3	>0.8	>0.8	>0.9	>0.9	>0.9
Research 2	2.443	0.830	0.877	0.924	0.912	0.923

**Figure 2:**
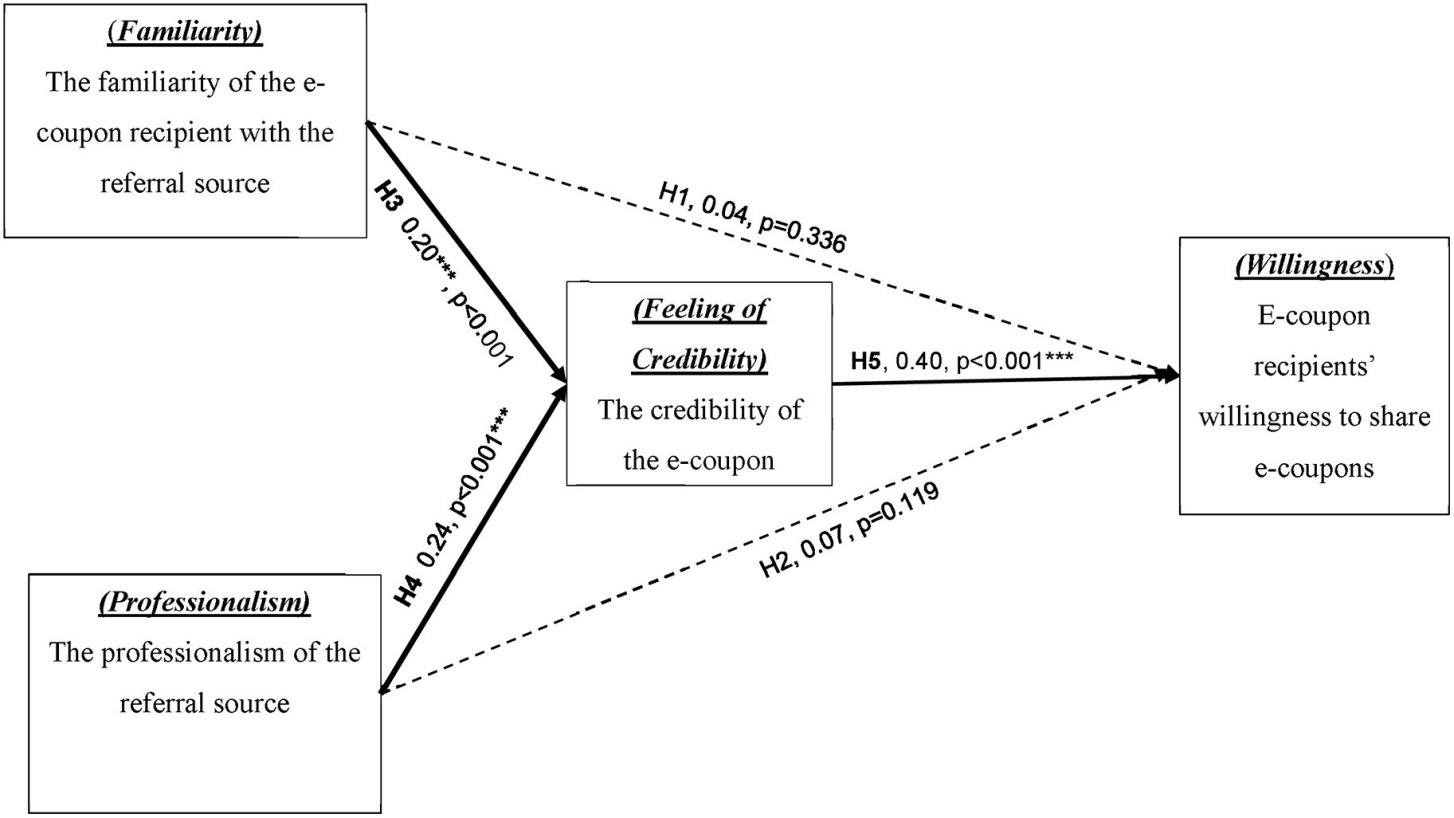
Hypotheses testing results of the research framework of this study.

Hypotheses testing results (Table 5) revealed the positive role of e-coupons in shared marketing. The results showed that familiarity (H3, 0.20, *p* < 0.001^**^) and professionalism (H4, 0.24, *p* < 0.001^**^) have significant positive impacts on the feeling of credibility, and the feeling of credibility has a positive effect on willingness to share (H5. 0.40, *p* < 0.001^**^). The test results of the SEM are shown and illustrated in Table 3 and Figure 2. These indicate that H3, H4, and H5 are supported. On the contrary, familiarity and professionalism did not have a significant and direct impact on recipients' willingness to share (H1, 0.04, *p* = 0.336; H2, 0.07, *p* = 0.119), indicating that both H1 and H2 are not supported.

**Table 5:** Research 1: model path coefficients and verification results.

**Hypothesis**	**Variables**		**Variables**	**Normalized** **path coefficient**		**Test results**
H1	Familiarity	→	Willingness to share	0.04	0.336	False
H2	Professionalism	→	Willingness to share	0.07	0.119	False
H3	Familiarity	→	Professionalism	0.20	***	True
H4	Professionalism	→	Professionalism	0.24	***	True
H5	Feeling of credibility	→	Willingness to share	0.40	***	True

*P ≤ 0.05; **P ≤ 0.01;

*** *P ≤ 0.001*.

## Discussion

Familiarity and professionalism were found to affect the feeling of credibility, increasing consumers' willingness to share and use e-coupons. This study's results on the impact of familiarity on the feeling of credibility and the impact of the feeling of credibility on willingness to share are consistent with the current market situations in the new century, in which e-commerce has obtained a dramatic and significant development where knowledge quantity is bursting.

Also, the research results of this study proved that the social sharing behavior theory can be applied as the theoretical basis for sharing marketing behavior with e-coupon sharing as an example.

### Regarding Professionalism of the Referral Source

Nowadays, consumers increasingly rely on the information from the Internet's word-of-mouth, including the reviews, comments, and recommendations of Internet celebrities, professionals, and product certification bodies (e.g., government, national, and international certification authorities) posted on social media sites. For example, every time a new product is launched on the market, several unboxing videos and blog articles made by different individuals can be found on Twitter, YouTube, Instagram, and other social media sites with the intent of sharing their knowledge and thoughts.

Consumers may be encouraged by the discount e-coupons offered by different companies when purchasing products; however, this is not the only consideration they consider. They also regard the quality of the products based on experts' reviews and if they are certified by a credible source. Further, even if peers or experts share the e-coupons in the field, consumers may only be willing to use the e-coupons and share them with others if the quality of the product has been certified by an expert; thus, the feeling of credibility of the source has to be high for e-coupons to work as a marketing strategy.

### Regarding Familiarity of the E-Coupon's Recipient With the Referral Source

When the referral source of the e-coupon is a friend, a family, or have a close relationship with the recipient, there is a high chance that the e-coupon will be used to purchase the product. This also indicates that the feeling of credibility exerts a great impact on the use and sharing of e-coupons.

This may be the case because of the importance of social relations in the current society. Today's era is characterized by an explosion of knowledge and increased speed of knowledge dissemination interspersed with frequent cases of Internet fraud. If the feeling of credibility is low, individuals who haphazardly share e-coupons with friends and relatives may be complained about and criticized. Therefore, if e-coupons have not been verified to be credible enough, individuals may not easily share e-coupons with others. On the other hand, if consumers are familiar with the e-coupon's referral source and if this individual is a credible source of knowledge and is trustworthy, they will be more willing to accept, use, and share e-coupons with other friends and family.

Finally, the author did not hypothesize that credibility could be a mediator of the conceptual framework. That is because consumers behaviors are quite complicated. This study verified four variables only to prove the conceptual relationships between social sharing behavior in terms of benefits and product. To prove credibility is a mediator, sample base and moderators and other influential factors' inferences should be expanded. Therefore, this study proposed testing credibility as the mediator in the suggestions for future studies.

## Conclusion

### Theoretical Implications: The Interplay Role of Products and Social Psychology in the Field of Social Marketing

**Figure 3:**
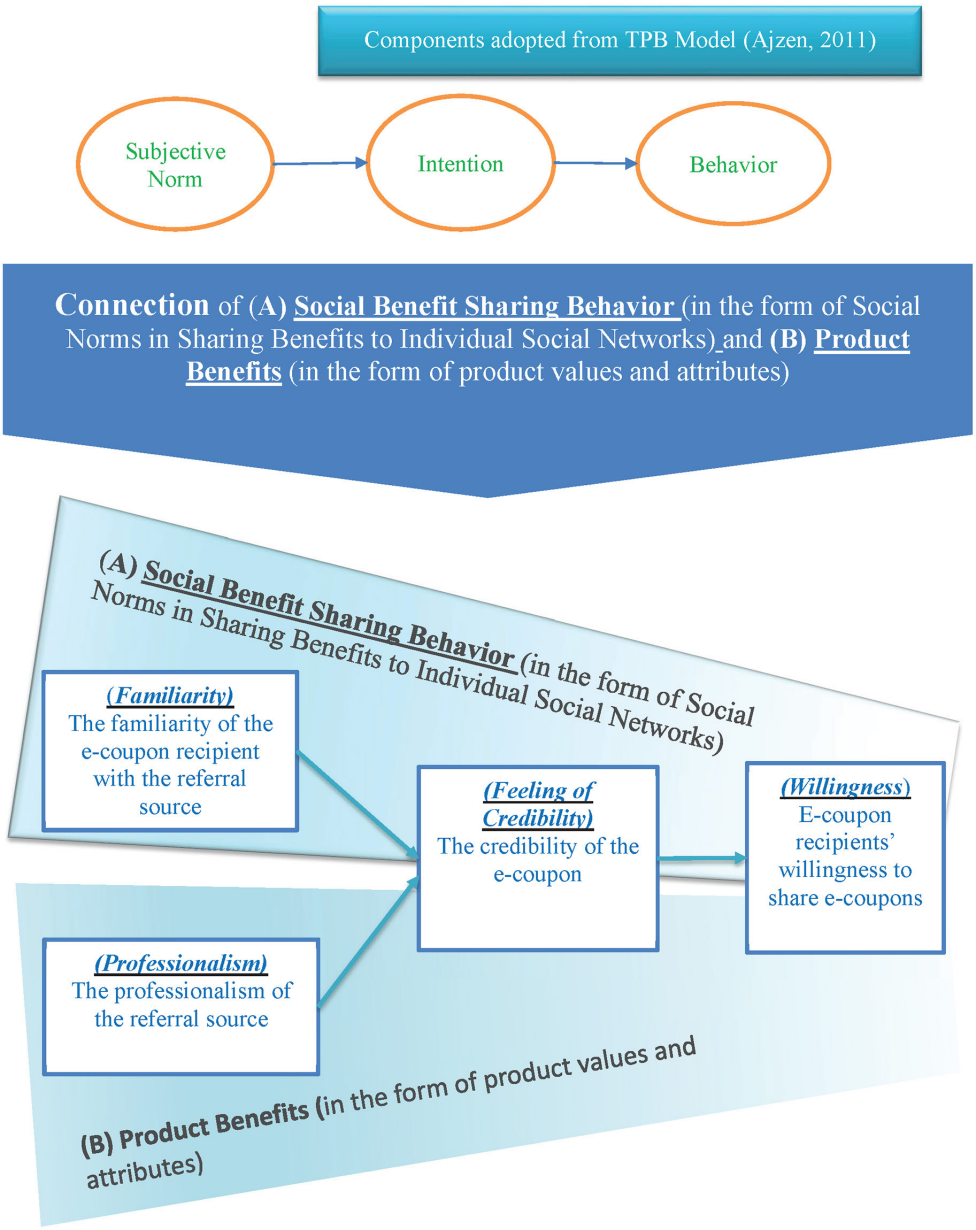
Connection mechanism between social benefit sharing behavior and product benefits (Source: this study).

As mentioned earlier, this study expected to adopt the perspective of social sharing behavior of consumers to extensively explain that the community-based social marketing is covered in the range of social norms - individuals in the society expects to share gained benefit to their social ties such as benefits in the form of advantageous products' values and attributes. Therefore, the author proposed a new conceptual model (Figure 3) to distinguish and clarify the interplay role of products and social psychology in the field of social marketing. It is hoped that such an extensive explanation can assist the further development and expansion of social marketing theory.

This study theoretically contributed to indicate the connection mechanism between (A) Social Benefit Sharing Behavior and (B) Product Benefits. From Figure 3, we can see that TPB Model mentioned that social norms influence individuals' intention and finally leads to behavioral results (Components adopted from Ajzen, 2011), which helps explain the mechanism for the connection between (A) social benefit sharing behavior (in the form of social norms in sharing benefits to individual social networks) and (B) product benefits (in the form of product values and attributes) as proposed by this study. From the bottom part of Figure 3, it is found that the three variables familiarity, feeling of credibility and willingness to shares are covered by the A mechanism (social benefit sharing behavior in the form of social norms in sharing benefits to an individual's social networks), and the other three variables professionalism, feeling of credibility and willingness to share are covered by the B Mechanism, the product benefits, in the form of product values and attributes).

### Managerial Implications

This study recommends that marketers endorse e-coupons through referral sources with higher familiarity and professionalism. By increasing the feeling of credibility in e-coupons, target consumers may be encouraged to use and share them. For example, companies may provide a 7-day money-back guarantee if the product is proven unsatisfactory. Consumers with a higher feeling of credibility on endorsed products and services may have a higher level of confidence in the e-coupons, and they may become the pioneer consumers in sharing discounted e-coupons with their relatives and friends.

When promoting e-coupons, this study suggests that marketers may provide incentives for peer-to-peer sharing. Thoroughly choosing the experts that will be tapped to recommend the e-coupons may also be a good strategy for sharing the e-coupons (i.e., a pharmacist with a professional image to recommend vitamins). It may also help if the referral source has a long-standing dependable relationship with fans and followers, as this will improve the credibility of the e-coupons and promote their sharing and actual use.

### Research Limitations

Research limits of this study source from (1) past investigations on the e-coupon behavior patterns based on credibility, professionalism, and familiarity are not much; therefore, some of the cited literature is kind of old. Although the authors have tried to input more recent outstanding research works into the literature, the authors believe that with the growth of research in this field, a literature review should be updated better then. (2) If possible, it is suggested a variety of verification of more numbers of research samples or samples from western cultures could be done to increase multiple tests on the validity of the research results. (3) The currently chosen methodology is kind of narrower than what is needed to support the broader conclusions of the work. Therefore, it is also suggested authors of future studies can choose a research method that is better in possibly supporting a broader conclusion of the research results.

### Suggestions for Future Studies

Based on the results of this study, the feeling of credibility is an important key variable to encourage consumers' purchasing behavior. Even if consumers have good relationships with their friends and family who may share them with e-coupons or if professionals recommend the e-coupons' products or services, the feeling of credibility has to be high for consumers to be willing to share and use the e-coupons. Because of this, future studies need to explore how consumers could gain confidence in the professional's recommendation or how relationship marketing could increase the credibility of the product or service. It is also suggested that future studies can verify if the variable of the feeling of credibility also serves to be a mediator for the distribution of e-coupons with more number of research objects and number of variables, especially moderators for testing.

## Data Availability Statement

The original contributions presented in the study are included in the article/supplementary material, further inquiries can be directed to the corresponding author/s.

## Author Contributions

AS: making sure the conceptual research framework of this study, confirmation and inferring of hypothesis, abstracts, introduction, supervising on the progress of the research, conclusions' theoretical and practical implications, and update and revision of the manuscript according to reviewers' valued comments including the newly added Figure 3. K-FC: inferring of hypothesis, statistical analysis, corresponding, checking, formation, submission, literature review update, update and revision of the manuscript according to reviewers' valued comments, and including the newly added Figure 3. Y-HH: data collection and analysis, reformation of the figures and tables, conclusion presentation regarding future studies and research limitations, translation, and update and revision of the manuscript according to reviewers' valued comments. All authors contributed to the article and approved the submitted version.

## Funding

This study was supported by the grant from LinNan Normal University; the fund number is 117/1170919327.

## Conflict of Interest

The authors declare that the research was conducted in the absence of any commercial or financial relationships that could be construed as a potential conflict of interest.

## Publisher's Note

All claims expressed in this article are solely those of the authors and do not necessarily represent those of their affiliated organizations, or those of the publisher, the editors and the reviewers. Any product that may be evaluated in this article, or claim that may be made by its manufacturer, is not guaranteed or endorsed by the publisher.
